# Tribological Behaviour of Enamel Coatings Created by a Prototype Device for Local Repair of Inorganic Surfaces

**DOI:** 10.3390/ma16031224

**Published:** 2023-01-31

**Authors:** Miroslav Müller, Monika Hromasová, Petr Valášek, Iva Nováková, Jaromír Moravec, Milan Jelínek

**Affiliations:** 1 Department of Material Science and Manufacturing Technology, Faculty of Engineering, Czech University of Life Sciences Prague, Suchdol, 16500 Praha, Czech Republic; 2 Department of Electrical Engineering and Automation, Faculty of Engineering, Czech University of Life Sciences Prague, Suchdol, 16500 Praha, Czech Republic; 3 Department of Engineering Technology, Faculty of Mechanical Engineering, Technical University of Liberec, 46117 Liberec, Czech Republic

**Keywords:** inorganic surface, enamel, wear, interaction, local repairs, abrasive water jet, SEM

## Abstract

The ability of materials to withstand environmental influences is a frequent necessity in many industries. Special requirements are imposed by such industries where surfaces are affected by acidity during the processing or storage of products. In such cases, when the basic surface is exposed to chemical influences, it is possible to use enamel coatings, which, with their properties, guarantee the protection of the surface and achieve the required service life of the material. This article deals mainly with the interaction between the base material and the enamel and its resistance to wear between the original and the renovated surface caused by local heating. The article presents a methodical procedure for the preparation of test specimens with an enamel layer prepared by AWJ cutting, eliminating its damage. There are minimal differences in the microstructure between the original and the renovated surface due to the production technique. The renovated enamel surface had more bubbles of a larger size than the original surface. Good adhesion between the base metal material (substrate) and the ground coat was demonstrated. The tested surfaces demonstrated high resistance to intensive abrasion conditions with low linear wear increments.

## 1. Introduction

Enamelled coatings are inorganic glass-based surfaces with a complex chemical composition that protect the base material from unwanted, especially chemical, influences. The advantages of enamelled surfaces can be seen in the optimized properties of thermal expansion and chemical inertness. Resistance to strong acids, abrasion, and high temperature is desirable in application areas where a smooth surface is simultaneously required [1,2,3]. Enamel coatings in aggressive environments have a significant effect on corrosion resistance in hot environments [4], for example, by preventing oxygen and chlorine from affecting the substrate. Concrete use can be found, for example, in enamelled pressure vessels in the chemical, oil, food, and pharmaceutical industries [5,6,7]. The enamel [8] on the surface of the materials resists abrasion and scratching, making the surface of the material generally able to withstand mechanical stress. Enamelled containers are part of complex processes in industries where, if a defect occurs in the enamelled surface, it is usually necessary to shut down the entire operation. One of the options for repairing such applications is local enamel replenishment. This method of repair—through targeted local heating—is preferred above all from the point of view of time consumption, when the repair can be carried out without disassembling the system, and it also has an obvious economic benefit.

The interaction between the underlying material and the enamel surface is crucial with respect to the durability of the enamel surface and its ability to protect the underlying material. The interphase interaction, the integrity of the enamel layer and its porosity can be described as a weak link, especially because of the possible leakage of the media to which the surface is exposed [9]. The interaction itself can be defined as a physicochemical reaction that occurs during firing and can be assessed by electron microscopy.

Mechanical characteristics, especially wear resistance [3], can be considered an important property. Resistance to abrasion and scratching is one of the important characteristics [8,10,11] of enamelled surfaces. It is important to reduce the porosity of the enamel, so that abrasion resistance [8,12] is optimal. Enamel coating has excellent properties, which include especially wear resistance [8], high hardness [13], chemical inertness [14], chemical resistance [14], etc. During tribological testing [15], enamel can protect metallic substrates from damage caused by severe mechanical impact. Enamelled surfaces [15] can significantly improve wear resistance up to several times compared to the underlying material. Excellent tribological properties [16] are the key to achieving the long-term reliability of functional surfaces. Due to a wide range of positive physicochemical and mechanical properties, enamel surfaces are used in various industries [8]. An important area is the food industry. Research shows [8] that enamel coatings suffer mainly from abrasive wear-ploughed grooves, distinct delamination, and spallation. The above is a consequence of the fragility [8] of enamelled surfaces. One option [17,18] for cutting hard material, if necessary, is to use a water jet. The advantage of waterjet cutting is that the area is not thermally affected, and no internal scouring occurs. These characteristics of waterjet cutting, if the integrity of the layers is maintained, can also be used for [17,18] enamelled surfaces.

The aim of this article is to assess the interaction between the base metal material and the enamel surface applied by local heating through electron microscopy. At the same time, the article describes three-point abrasion, i.e., the wear resistance of this layer, when it is necessary to verify that the enamel applied by local heating shows greater resistance to abrasion than the basic material and its resistance is comparable to the surrounding—original—enamel layer. Research activities are connected with the verification of the implementation of the technology of local repairs of the integrity of inorganic (enamel) surfaces. The possibility of renovating locally damaged enamel surfaces, e.g., tanks, storage tanks, and mixers, reduces the cost of purchasing new equipment and the associated waste production in the form of damaged equipment. The contribution of the research is the partial measurements leading to the verification of the functionality of the innovative technology for the renovation of enamelled surfaces.

## 2. Materials and Methods

The research presented in this article was based on partial research activity within the solution of the MPO project Production of a prototype device for local re-enamelling, including feedback control and verification of its functionality (MPO FV 40144), the main goal of which was to create a prototype of a mobile device enabling local repairs of functional inorganic (enamelled) surfaces of reservoirs, pressure tanks and mixers and the related methodology for repairing these surfaces.

Specific tests verifying the functionality of the prototype device are the assessment of adhesion and homogeneity using SEM cross-sections and abrasive wear of the surface. For the preparation of the tested samples, it was necessary to prepare a sampling methodology using abrasive water jet technology, which would eliminate the violation of the integrity of the new inorganic surface. The tests were carried out on the basic compact and repaired surface.

### 2.1. Material of Research and Preparation of Test Specimens

The test samples were prepared on the HF-25kW-2022 prototype device intended for local repairs of enamel surfaces. Thanks to the frequency of 300 kHz, the device performs induction heating of thin surface metal layers. Thanks to the generator power of 25 kW and feedback control, the device enables local re-enamelling at temperatures up to 960 °C with freely selectable heating and cooling speeds. Both the device and the repair procedure are patented.

The local repair procedure was carried out on enamelled plates measuring 350 × 350 × 12 mm. Enamel with a thickness of approx. 1.38 mm was applied to the test board in the standard way in four layers (2× ground and 2× cover enamel). A circular defect with a size of 300 mm^2^ was ground on the center of the plate. The thickness of the applied enamel layer was checked using a PosiTector 6000 thickness gauge, with an average unfired enamel thickness of 0.563 mm. During the experiments, the firing temperature was measured using a pyrometer.

Based on previous experiments, the following technological parameters were evaluated as optimal for local repair: The following parameters were set for firing the first layer of repair enamel—temperature 880 °C, heating rate 50 °C·min^−1^, duration at temperature 1 min and cooling rate 50 °C·in^−1^. In the next step, a second layer of repair enamel was applied, which was fired at a temperature of 850 °C, a heating rate of 50 °C·min^−1^, a temperature hold of 1 min and a cooling rate of 50 °C·min^−1^. The first layer of covering enamel was then applied to the two layers of repair enamel, which was fired at a temperature of 830 °C, a heating rate of 50 °C·min^−1^, a temperature hold of 10 min and a cooling rate of 50 °C·min^−1^. A second layer of cover enamel was applied to the first layer of cover enamel, which was fired at the same heating and cooling rates, but at a temperature of 820 °C for 10 min.

When testing the quality of inorganic surfaces, it was necessary to ensure effective preparation of test specimens, i.e., sections of delivered samples, while damage to the applied inorganic layer had to be prevented. Several procedures were chosen. The resulting procedure eliminated damage to the applied inorganic layer. Therefore, it was first necessary to carry out sampling, which is not possible with the usual methods of material division. Original enamel and renovated enamel applied by local heating was “blue enamel” type G3084/SB RM 3 (producer Wendel GmbH). This type of enamel is suitable for contact with food and complies with the requirements of Regulation (EC) No 1935/2004 of the European Parliament and the Council, as amended by the following regulations and Decree No 38/2001 Coll. of the Ministry of Health of the Czech Republic.

When using conventional methods [19,20,21,22] of dividing materials with different coatings and surface treatments, they can be damaged, for example, by delamination, especially due to the contact of the tool with the workpiece. Research has shown that it is appropriate to use an unconventional machining method using water jet technology [20,23,24] characterized by considerable flexibility of adjustable machining parameters. However, it is necessary to take into account the optimization of the cutting process for a specific application. The primary cuts were made using AWAC abrasive water jet technology, characterized by pushing water through a water jet under high pressure while the secondary acceleration of the water jet to high speed occurs. The supplied plate was cut using AWAC CNC AWJ CT 0806 (AWAC, s. r. o., Prague, Czech Republic, using abrasive water jet (AWJ)) technology, and a plate was taken from the repair side for both metallographic evaluation and abrasive tests. The abrasive added to the waterjet for the AWJ technology was Australian GARNET MESH 80, which was added into the waterjet by the Bimba Flat at a mass flow rate of 442 ± 75 g × min^−1^. Several feed rates of the cutting head were evaluated: 5, 10, 20, 30, 40 and 50 mm·min^−1^. The results of the research showed that the optimal feed of the cutting head is 50 mm·min^−1^. The main evaluation criterion was the elimination of damage and delamination of the enamel layer. The following parameters were constant, i.e., working water pressure 380 MPa, focusing tube diameter 0.8 mm, the distance of the nozzle above the material to be cut 5 mm, and the angle of the nozzle above the material to be cut 90°. The samples intended for SEM analysis were subsequently cut using a metallographic cutter Struers Discotom 10 (Struers LLC, Cleveland, OH, USA) with a cutting disc for hard materials, the subsequent casting of the samples, grinding and polishing of the surface on Struers Tegramin 30 (Struers LLC, Cleveland, OH, USA).

**Figure 1:**
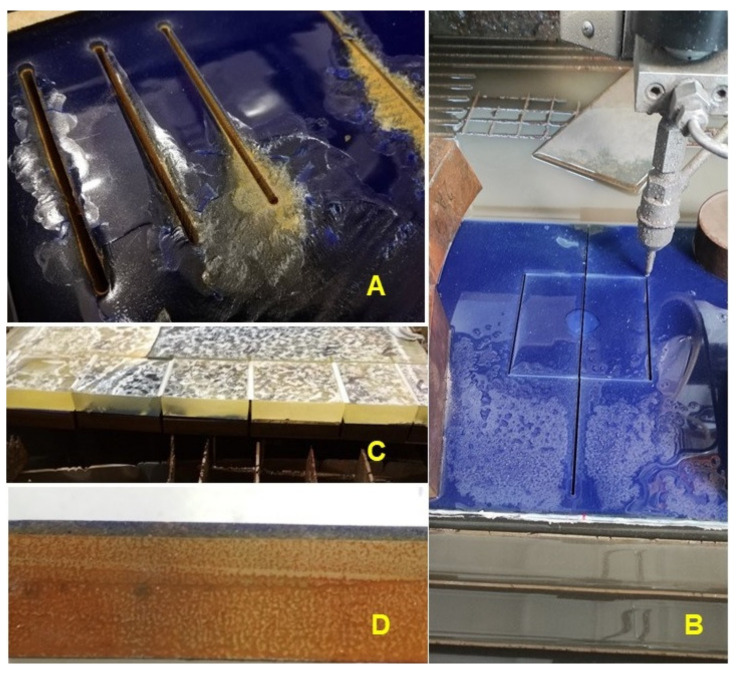
Preparation of test specimens—sampling from the panel after local treatment of the inorganic surface: (**A**): damage to the inorganic surface during sampling at different cutting parameters by AWJ technology, (**B**): cutting by AWJ technology of test specimens with a protective layer of two-component laminating epoxy resin on the functional surface, (**C**): a cross-sectional view of the test panel—sandwich layer—metal, inorganic surface, laminating epoxy resin layer, (**D**): test specimen after removing the separation layer with a protective layer of two-component laminating epoxy resin on the functional surface.

The research showed that even when using the abrasive water jet method, there is considerable damage to the inorganic surface (Figure 1A). Huynh et al. [8] state that enamelled surfaces show high resistance to wear but are characterized by high brittleness. This problem was solved by applying an approximately 20 mm layer of two-component laminating epoxy resin to the functional surface (Figure 1B,C). This resulted in a sandwich panel that eliminated damage to the inorganic layer during the preparation of test specimens during their machining (Figure 1C). At the place of wear testing, a separating fabric was inserted between the inorganic layer and the cover layer of the two-component laminating epoxy resin, thereby preventing the formation of adhesive bonds between the layer of laminating epoxy resin and the functional surface (Figure 1D).

**Figure 2:**
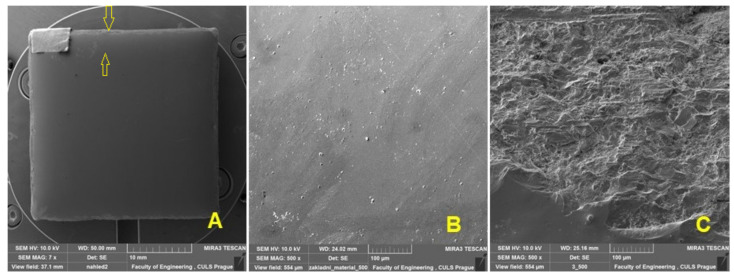
SEM analysis of the surface before wear: (**A**): produced test specimen (MAG 7×), (**B**): macroscopic view of the enamel surface (MAG 500×), (**C**): surface texture visible after the cut made with the AWJ technology (MAG 500×.).

Figure 2A shows the shape of the test body intended for abrasive wear testing and, at the same time, the partial positions of the testing using SEM analysis. Figure 2B shows the enamelled surface on which the abrasive resistance testing of the new functional surface was performed (position: base material). From Figure 2C, the extreme part of the enamelled surface marked with position No. 1 can be seen with a prominent trace after machining using AWJ technology.

### 2.2. SEM Analysis

Qualitative parameters were evaluated using SEM (scanning electron microscopy) on a TESCAN MIRA 3 GM (Tescan Brno s.r.o., Brno, Czech Republic) microscope and a Quorum Q150R ES (Tescan Brno s.r.o., Brno, Czech Republic)—Sputtering Deposition Rate using gold. Prepared sections of test samples for testing the quality of inorganic surfaces using SEM analysis were dusted with 5 micrometres of gold. The parameters of the SEM images can be seen from the lower description part of Figures 4–8 at HV 10 kV with the use of SE and BSE detectors.

### 2.3. Tribological Evaluation of the Abrasive Resistance of the Base and Repaired Enamel Surface

Wear research was carried out in accordance with the Gost standard 23.208-79 [25] (Standard Test Method for Measuring Abrasion Using the Dry Sand/Rubber Wheel Apparatus) on test specimens intended for the abrasive test. It was used for research test equipment Tester T-07, ITC PIB Institution of Technology Radom and the BT-16 Controller (Instytut Technologii Eksploatacji, Radom, Poland).

**Figure 3:**
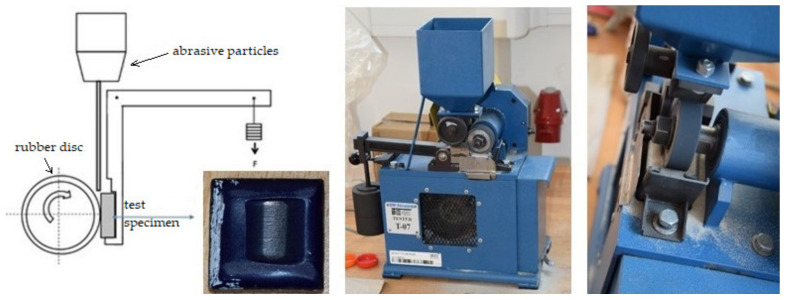
Representation of the device with a rubber disc and the test body.

In total, 150 cycles were tested, with one cycle representing the contact of the sample with a rubber disk at about 150 m. The test of resistance to abrasive wear was performed according to the ASTM G65 standard, i.e., on a device with a rubber disc (Figure 3). Sand particles with a size of 0.2 to 0.315 mm fell between the test body and the rubber disc. The sample load was 1600 g. The abrasive was floated Otava sand with a fraction size of 0.2 to 0.315 mm (according to the standard). Before and after each test (cycle), the test specimens were cleaned in an ultrasonic bath and dried with hot air. Mass losses were measured with a resolution of 0.1 mg on a Kern ABS analytical balance with a maximum mass of 120 g and an accuracy of 1.5 mg. Each measurement was repeated 3 times. Figure 3 also shows the test body and the worn area of the enamel surface.

## 3. Results

**Figure 4:**
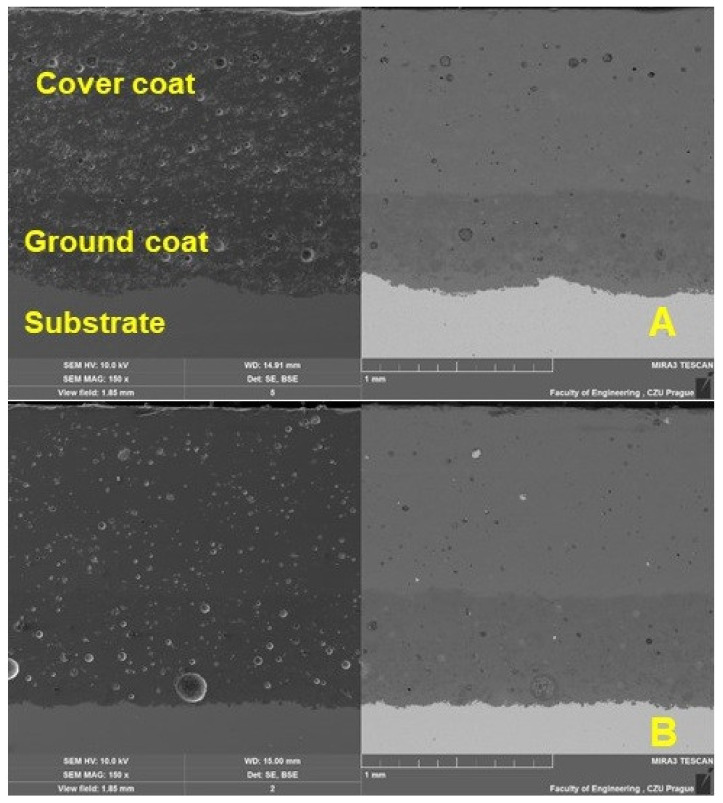
SEM analysis of a section of the test specimen before wear (SE Oxford detector—left, BSE Oxford detector—right): (**A**): original enamel (MAG 150×), (**B**): renovated enamel applied by local heating (MAG 150×).

Characterization of the coating was performed using SEM. The results of the SEM analysis shown in Figure 4 show a cross-section of the original and renovated enamel surface characterized by good interaction of the individual layers and significant porosity. The thickness of the ground coat of the original enamel was 482.72 ± 34.35 µm and of the renovated enamel applied by local heating was 577.25 ± 23.40 µm. The thickness of both surfaces differed slightly, by approx. 16%. The thickness of the cover coat of the original enamel was 955.39 ± 14.67 µm and of the renovated enamel applied by local heating was 973.97 ± 13.96 µm. The thickness of both surfaces differed slightly, by approx. 2%.

Figure 4 shows a section of a test sample intended for abrasive wear. Figure 4A shows a cross-section of the test body with the original enamelled surface, and Figure 4B shows a cross-section of the renovated enamelled surface created by induction local heating. From Figure 4A,B, three basic areas are visible (description from the bottom of the picture), i.e., the basic metal material—the substrate, the transition layer formed by the basic enamel fused directly to the metal and the top layer formed by the functional (covering) enamel. The fourth layer is the potting compound, without which it would be impossible to create a high-quality cut without damaging the examined enamel surface.

The inorganic enamel surface is characterized by considerable porosity. From the cross-sections presented on SEM, Figure 4, it can be seen that the bubbled structure shows both original and renovated enamel deposited by local heating. The bubbled structure shows many small but also many large bubbles.

**Figure 5:**
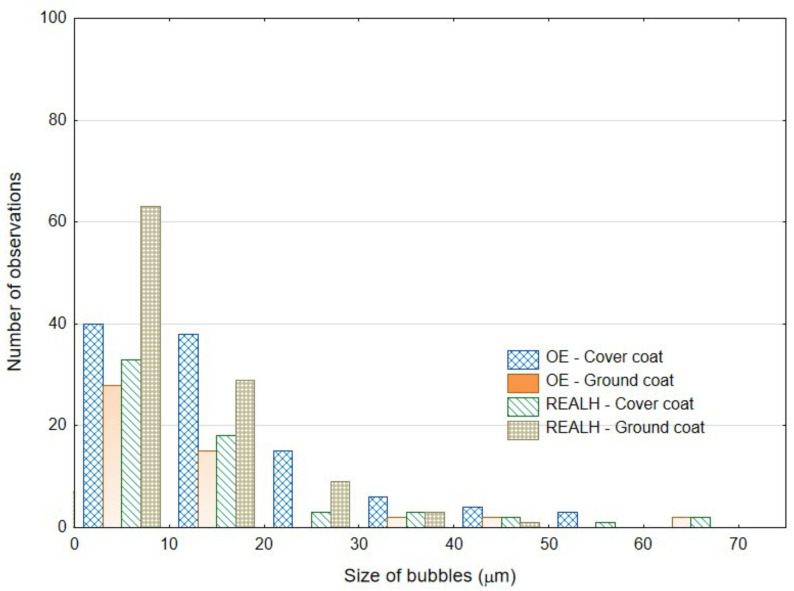
Bubble size in the enamel layer (Original enamel—OE, Renovated enamel applied by local heating—REALH).

Huynh et al. [8] present in their article findings from optical microscope image analysis. They point out that enamel coatings have bubble structures of different sizes [8]. These bubbles form near the base metal material (substrate) and increase in size towards the top layer. Huynh et al. [8] state that most bubbles are smaller than 15 µm, although some reach 20–40 µm in size and a few reach 50 or 80 µm in size. Huynh et al. [8] further state from the findings of their research that there are also bubbles in the functional covering layer, but they are smaller than in the base layer. The results of the bubbles in the cover coat and ground coat of the original enamel and the renovated enamel applied by local heating can be seen in Figure 5, except for one bubble with a size of 142.43 µm in the ground coat of renovated enamel applied by local heating—REALH, which was outside the measured range. The results of the coats tested by us confirm the results of Huynh et al. [8].

**Figure 6:**
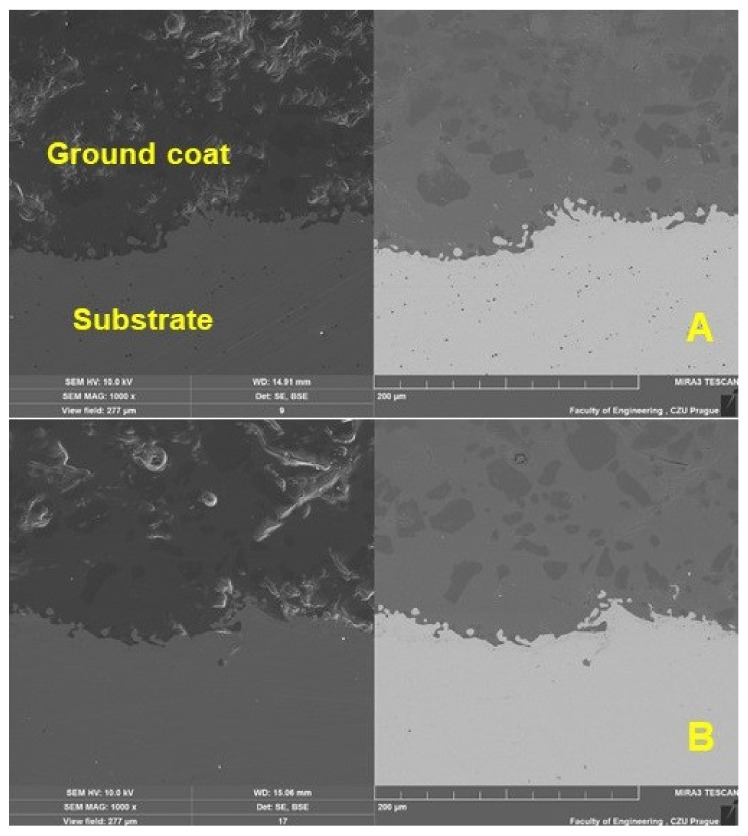
SEM analysis of the cross-section of the test specimen before wear—the interface metal material (substrate) and the base enamel surface fused to the metal surface (SE Oxford detector—left, BSE Oxford detector—right): (**A**): original enamel (MAG 1.0k×), (**B**): renovated enamel applied by local heating (MAG 1.0k×).

**Figure 7:**
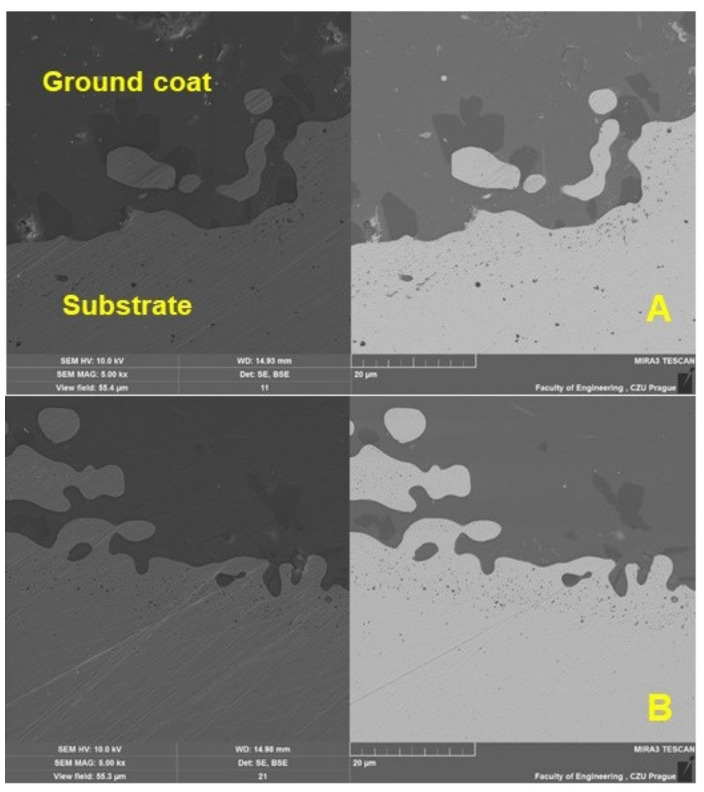
SEM analysis of a cross-section of a test specimen before wear—a detailed view of the interaction between the metal material (substrate) interface and the base enamel surface fused to the metal surface (SE detector Oxford—left, BSE detector Oxford—right): (**A**): original enamel (MAG 5.0k×), (**B**): renovated enamel applied by local heating (MAG 5.0k×).

Figure 6 and Figure 7 show a detailed view of the transition area, i.e., the interface of the metal material (substrate) and the base enamel surface fused to the metal surface. The pictures show a good interaction between the base metal material and the enamel surface, original and applied during the renovation by local heating. Figure 7 shows a detailed view of the mutual interaction between these surfaces. The ground coat usually has a low viscosity to allow better wetting of the base metal material [8]. It, therefore, aims to connect the basic metal material (substrate) with a functional cover coat. For this reason, the formation of bubbles at the interface between the metal material (substrate) and the base enamel fused to the metal surface should be limited. It is clear from Figure 7A,B that bubbles do not occur directly in the interface area.

**Figure 8:**
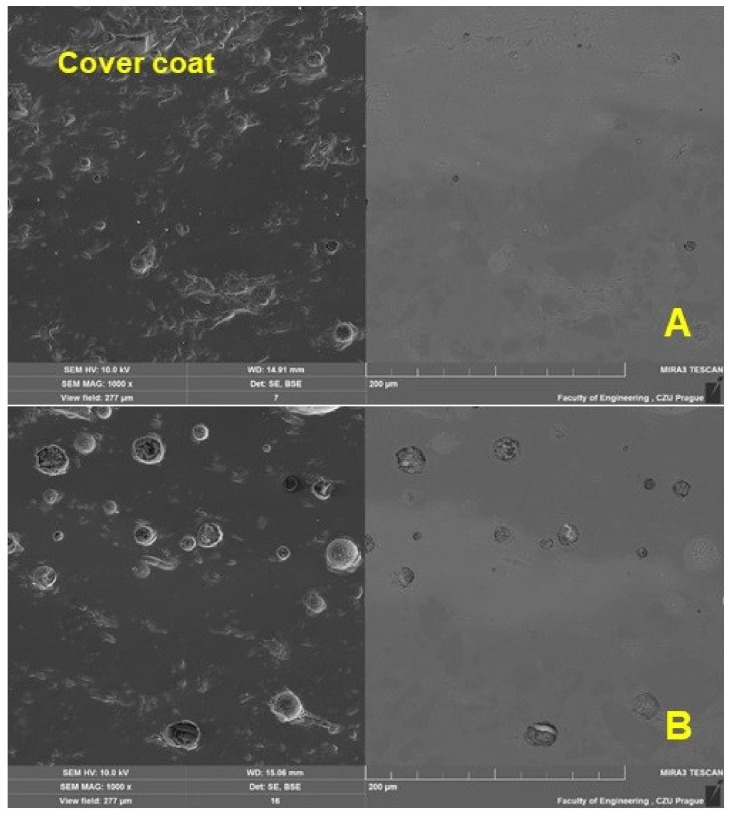
SEM analysis of the section of the test specimen before wear—a detailed view of the top (covering) enamel layer (SE Oxford detector—left, BSE Oxford detector—right): (**A**): original enamel (MAG 1.0k×), (**B**): renovated enamel applied by local heating (MAG 1.0k×).

Figure 8 shows the top functional (covering) enamel layer, which was quite porous. From Figure 8A,B, a comparison between the original and the renovated enamel surface applied by local heating is possible.

Abrasion tests were performed using the Standard Test Method for Measuring Abrasion Using the Dry Sand/Rubber Wheel Apparatus in accordance with GOST 23.208-79 [25].

**Figure 9:**
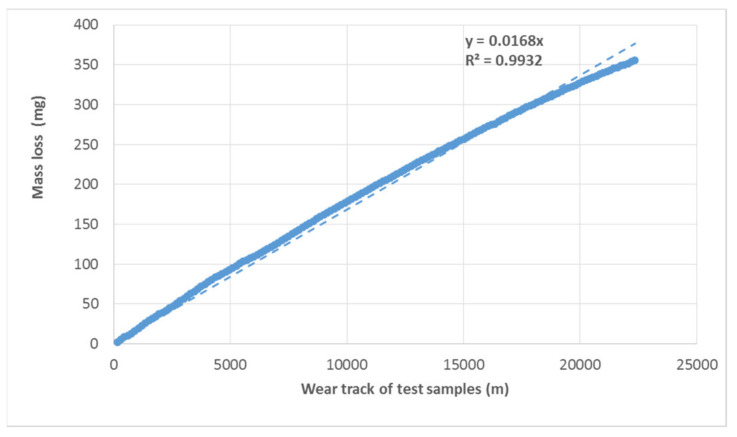
Course of wear of the tested material (mass loss is cumulative depending on the track) under a load of 1600 g.

**Figure 10:**
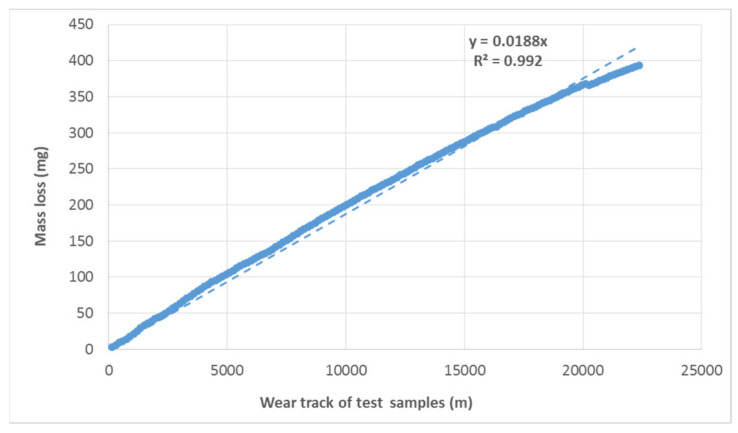
Course of wear of the tested material (mass loss is cumulative depending on the track) under a load of 1600 g on a renovated enamelled surface.

The course of wear shows a linear trend. The results show a linear increase in the mass loss of the tested materials depending on the wear track (Figure 9 and Figure 10). Baptista et al. [26] state that the volume of worn material is increasing slowly but consistently. These conclusions of Baptista et al. [26] are consistent with the research conclusions evident from Figure 9 and Figure 10. Tribological tests show a reduction in wear resistance of approx. 12% on the refurbished enamel surface applied by local heating. The linear trend of increasing mass loss is maintained. Only the direction of the trend given by the functional equation shown in the graph is slightly different. For a proper evaluation, it is also important to determine the determination index R^2^ by taking a value between 0 and 1. If R^2^ is equal to or close to 1, a significant correlation exists in this sample. The results show a strong dependence, i.e., there is no difference between the calculation and the actual measured values.

**Figure 11:**
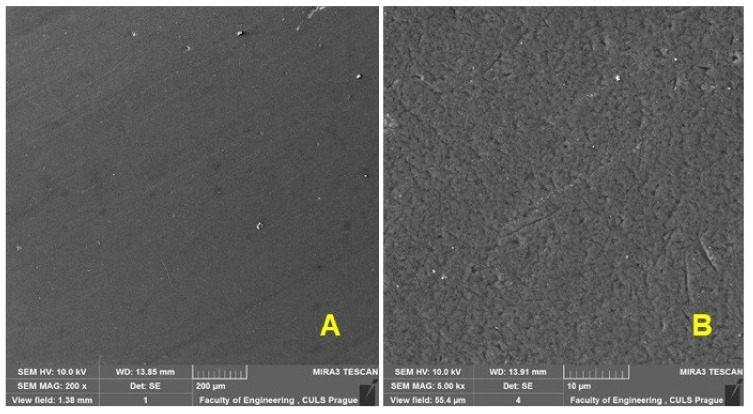
SEM analysis of the surface before wear: (**A**): a macroscopic image of the surface (MAG 200×), (**B**): a detailed image (MAG 5.0k×).

**Figure 12:**
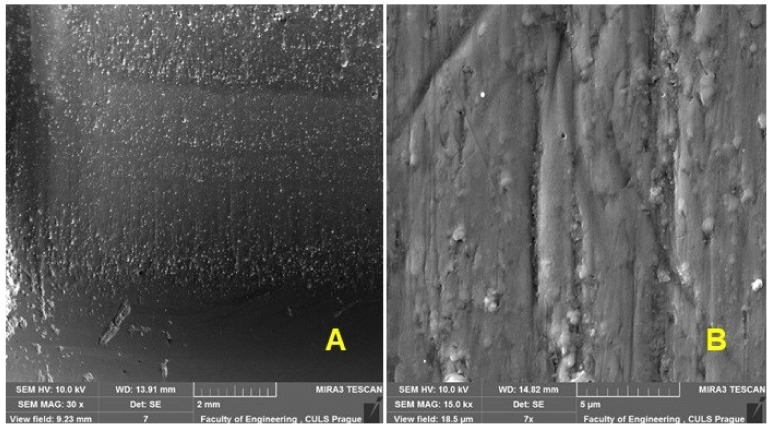
SEM analysis of the surface—the leading surface of the rubber disc during the enamel surface wear process: (**A**): a macroscopic image of the surface (MAG 30×), (**B**): a detailed image of the surface (MAG 15.0k×).

In order to evaluate the wear process of the renovated enamel surface, SEM evaluation of the worn area of the enamel layer in different places was performed. Figure 11 shows an enamel surface that has not been exposed to wear. Figure 12A shows the transition surface between the original (right) and worn (left) surfaces of the test specimens. Figure 12B shows a significant mark on the surface after the action of abrasive particles.

**Figure 13:**
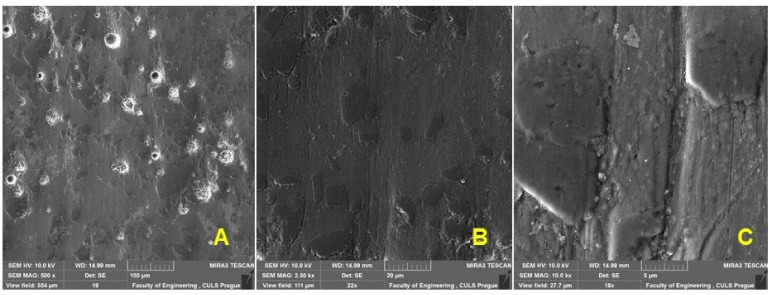
SEM analysis of the surface—the central part of the wear mark, in which intense wear acted on the enamel surface: (**A**): a macroscopic image of the surface (MAG 500×), (**B**): a detailed image of the surface (MAG 2.5k×), (**C**): a detailed image of the surface (MAG 10.0k×).

Figure 13 shows the SEM analysis of the central part of the wear mark, in which intensive wear has affected the enamel surface. Figure 12A shows the morphology of wear marks after tribological tests, where the exposed bubbles contained in the cover coat are visible. Huynh et al. [8] present in their results a wear track using SEM analysis, which shows the morphology of the wear tracks of the coatings after tribological testing [8] when the surfaces of all coatings were covered with exposed bubbles. Similar results can be seen in Figure 13. In the central part, the tracks of a rubber disc (Figure 13B,C) are visible, which causes intensive wear with abrasive particles (free abrasive) on the enamel surface. The good integrity of the enamel surface is evident. Figure 13 shows tracks of abrasive particles pressed against the surface with a rubber disc on the worn enamel surface.

A detail of a crack creation at a micro fatigue mechanism at the enamel surface wear is obvious from Figure 12B and Figure 13C. Micro fatigue is connected with a mechanism of worn material removal at the abrasive wear called enamel surface when a new abrasive particle acts on the worn material after the groove creation and causes a new deformation in the already deformed area of the enamel surface.

Enamel coatings suffer mainly from abrasive wear with ploughed grooves, obvious delamination and flaking. These findings of Huynh et al. [8] were confirmed.

Huynh et al. [8] reported that the scratch comparison shows that the enamel coating was resistant to heavy wear. They further indicated that the zircon phase also provided wear resistance to the enamel coating by improving its hardness, but the enamel with a pure glass phase caused severe abrasive wear due to its high brittleness and lower fracture toughness [13]. Wang points out that chemical composition and structural analysis of the worn surfaces reveal tribological [13] differences between the different enamel coatings.

Table 1 shows the microhardness values HV0.05 of a cross-section of the test body with the original enamel and renovated enamel applied by local heating. The results show a minimal difference in the measured values between the original enamel and renovated enamel applied by local heating, varying in the interval from about 0.45 to 2.05%. Wang [13], in his research, states that the functional layer of enamel coating on steel substrate had up to 991 HV0.05. A more pronounced difference was in the microhardness measurements of the base-steel substrate, where the renovated enamel applied by local heating increased the microhardness HV0.05 by up to about 17.5%.

**Figure 14:**
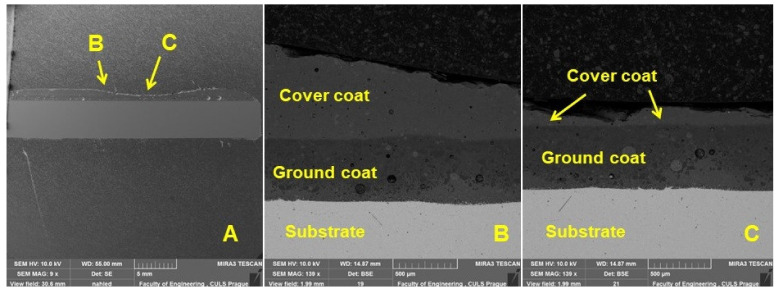
SEM analysis of the section of the test specimen after wear: (**A**): longitudinal section of the tested specimen after wear (MAG 9×, (**B**): longitudinal section in the area of the leading surface of the rubber disc during the wear process of the enamel surface (MAG 139×), (**C**): longitudinal section in areas of the central part of the worn enamel surface (MAG 139×).

To evaluate the wear process of the enamelled surface, SEM evaluation of the longitudinal section in different places of the wear mark was performed (Figure 14). Figure 13 A shows a cross-section of the worn surface, from which the mark created after the end of the 150th measurement cycle is evident. Figure 14B presents the leading (marginal) surface of the wear. Figure 13C shows the central part of the worn enamel surface, where intense wear occurred. It is also clear from Figure 14C that after 150 cycles (22,350 m), almost the entire top layer of the enamel surface was removed.

## 4. Conclusions

The article presents the results of the interaction between the base metal material and the enamel surface applied by local heating through electron microscopy. The enamel layer renovated with a prototype device using local induction heating is evaluated from the point of view of resistance to abrasive wear Method for Measuring Abrasion Using the Dry Sand/Rubber Wheel Apparatus. The stated results bring new knowledge in the field of enamelled surface renovation using local induction heating, which is beneficial for practical application, given the potential for application in large containers, for example, used in the food industry. A significant benefit can be seen in the testing methodology, i.e., in particular, the preparation of test samples using a sandwich structure that eliminates damage to the fragile enamel coating.

SEM analysis of the cross-sections of the coatings showed minimal microstructural differences due to the production technique, i.e., between the original and the refurbished enamel surface applied by local heating. The enamel coating is characterized by a bubble structure of different sizes. There were more bubbles in the renovated enamelled surface, especially in the ground coat. There was also a greater representation of larger bubbles. The detailed results of the SEM analysis showed good mutual adhesion between the base metal material (substrate) and the ground coat.

The tested enamelled surfaces demonstrated high resistance to conditions of intense abrasion, i.e., a linear increase characterized by small mass losses of the material. During the wear process, characteristic grooves created by interaction with sand particles with a size of 0.2 to 0.315 mm were formed on the surface.

## Figures and Tables

**Table 1:** Microhardness measurement HV0.05 (10 s load) for original enamel and renovated enamel applied by local heating.

Place of Measuring	Substrate	Ground Coat	Cover Coat
Original enamel	140.60 ± 2.29	763.10 ± 23.67	845.87 ± 8.49
Renovated enamel applied by local heating	165.13 ± 9.00	778.83 ± 23.37	849.57 ± 17.64

## Data Availability

Data sharing is not available.
